# Long noncoding RNA SNHG1 promotes breast cancer progression by regulating the miR-641/RRS1 axis

**DOI:** 10.1038/s41598-024-52953-0

**Published:** 2024-02-08

**Authors:** Lin Deng, Jun Wang, Junying Song, Qinglan Wu, Zunshuang Gong, Jinlian Song, Lin Hou

**Affiliations:** 1 Department of Biochemistry and Molecular Biology, School of Basic Medicine, Qingdao University, Qingdao, 266071 China; 2 Wanzhou District Center for Disease Control and Prevention, Chongqing, 404100 China; 3 WeiFang Maternal and Child Health Hospital, Shandong province, 261000 China; 4 Weifang Medical University Pediatrics Research Institute, Shandong province, 261000 China; 5 Wuhan University School of Basic Medical Sciences-Weifang Children’s Neurological Diseases and Innovation Transformation Joint Research Center, Shandong province, 261000 China; 6 Department of Laboratory, The Affiliated Women and Children’s Hospital of Qingdao University, Qingdao University, Qingdao, 266071 China

**Keywords:** RNA, Breast cancer, Metastasis, Oncogenes

## Abstract

An increasing number of studies have indicated the crucial involvement of long non-coding RNAs (lncRNAs) in the onset and progression of malignancies. However, a complete understanding of the molecular mechanism underlying the effect of abnormally expressed lncRNAs on breast cancer (BC) remains elusive. This study aimed to elucidate the influence of the lncRNA small nucleolar RNA host gene 1 (*SNHG1*) on BC progression and its underlying mechanism. Our findings revealed a conspicuous up-regulation of *SNHG1* in both BC tissues and cells. The downregulation of *SNHG1* was observed to inhibit BC cell proliferation, migration, invasion, and epithelial-mesenchymal transition (EMT) processes, while simultaneously promoting apoptosis. Furthermore, dual-luciferase reporter gene and RNA pull-down assays established that *SNHG1* targeted *miR-641* expression, while *miR-641* targeted RRS1. Rescue studies demonstrated that in vitro* SNHG1* silencing could be reversed by the *miR-641* inhibitor, as well as by *RRS1* upregulation. Moreover, in vivo downregulation of *SNHG1* was found to inhibit BC growth. Through the inhibition of the *miR-641* level, *SNHG1* elevated the level of the downstream target *RRS1*, thereby fostering BC growth, migration, and invasion while inhibiting apoptosis. These findings suggest that *SNHG1* may represent a potential therapeutic target for BC treatment.

## Introduction

Breast cancer (BC) stands as the most prevalent malignant tumor globally, serving as the primary cause of death among women^1^. Studies indicate that BC incidence and mortality rank highest among female malignant diseases in China^2^. BC, a heterogeneous disease originating from the ductal epithelium of the breast, correlates with factors such as pregnancy, hormone therapy, gender, age, and family history^3,4^. Despite the three main treatment modalities—radiation therapy, chemotherapy, and surgery—the recurrence rate of BC remains high, and the 5-year survival rate remains less than encouraging^5^. Consequently, understanding the molecular mechanisms of BC and identifying novel therapeutic targets is of paramount importance for improving patient outcomes.

Long non-coding RNAs (lncRNAs), defined as non-coding RNAs exceeding 200 nucleotides without protein-coding capability^6^, play vital roles in diverse biological processes, including protein translation modification, post-transcriptional regulation, transcription, and epigenetic modifications^7,8^. Increasing evidence suggests the involvement of lncRNAs in mediating BC processes^9,10^. For instance, the upregulation of lncRNA *RBM5-AS1* in BC promotes metastasis through hypoxia-induced RUNX2 transcriptional activation^11^. Xue et al. also found that *HOTAIR* overexpression affects BC cell proliferation and induces tamoxifen resistance in patients with BC^12^. LncCCAT1 influences the function of BC stem cells and promotes BC progression^13^. *SNHG1*, acting as an oncogene, is highly expressed in various cancers^14^. For example, *SNHG1* contributes to the progression of colorectal cancer by interacting with EZH2 and miR-154-5p^15^, whereas inhibition of *SNHG1* reduces prostate cancer cell growth and metastasis^16^. However, the specific molecular mechanism of *SNHG1* in BC remains unclear.

MicroRNAs (miRNAs), small non-coding RNAs ~ 22 nucleotides in length, regulate target gene expression by binding to the 3'-untranslated region (3'-UTR) of corresponding mRNAs^17^. Down-regulation of EVA1A by miR-103a-3p promotes hepatocellular carcinoma cell proliferation and migration^18^. In glioma, *miR-641* acts as a tumor suppressor by targeting E2F6 to inhibit cell proliferation and invasion^19^. The regulator of ribosome synthesis 1 (*RRS1*) functions as an oncogene in various tumors and plays a crucial role in BC occurrence^20^.

This study evaluated the impact of downregulating *SNHG1* on BC cell function in vitro and in vivo. Our findings indicate that *SNHG1* affects the proliferation, migration, invasion, and apoptosis of BC cells through the *miR-641*/*RRS1* axis, offering valuable insights into the pathogenesis of BC.

## Materials and methods

### Clinical samples

Tumor samples and adjacent fresh tissue samples were collected from 21 pairs of patients with BC at the Affiliated Hospital of Qingdao University. All clinical specimens were pathologically certified, and patient tissues were stored at − 80 °C. The study received approval from the Ethics Committee of the Medical College of Qingdao University (approval number QDU-HEC-2022259, date: November 1, 2022). All procedures adhered to relevant guidelines and regulations, and informed consent was obtained from all participating patients.

### Cell culture

The human normal mammary epithelial cell line MCF-10A (SCSP-575) and human BC cell lines BT-549 (TCHu93), MDA-MB-231 (TCHu227), MCF-7 (SCSP-531), and MDA-MB-468 (TCHu136) were provided by the Chinese Academy of Sciences (Shanghai, China). Cells were cultured in a 37 °C incubator with 5% CO_2_. Before experiments, all cell lines were validated and tested negative for mycoplasma. MCF-10A cells were cultured in DMEM/F12 (Hyclone, Seattle, WA, USA), while BC cell lines were grown in Dulbecco’s modified Eagle medium (DMEM) with 10% fetal bovine serum (FBS; Shanghai Excel Biology, China) and 1% penicillin–streptomycin solution.

### Quantitative real-time polymerase chain reaction (qRT-PCR)

Total RNA was initially extracted from BC cells and tissues using the TRIzol reagent (Invitrogen, Waltham, MA, USA), and RNA quantity was determined by spectrophotometry. The HiScript III All-in-one RT SuperMix kit (Vazyme, Nanjing, China) was utilized to convert RNA to cDNA. For miRNA, the miRNA first strand cDNA synthesis kit (Vazyme) employed the stem-loop method. qRT-PCR was performed using the ChamQ Universal SYBR qPCR Master Mix (Vazyme). The relative abundance of RNA genes was calculated using the 2^−ΔΔ^Ct method. The list of primers used is provided below.

**Table:** 

GAPDH Forward primer	5′-AACGGATTTGGTCGTATTGGG-3ʹ
GAPDH Reverse primer	5′-TCGCTCCTGGAAGATGGTGAT-3ʹ
SNHG1 Forward primer	5′-ACAAGAGCTTACTGGTGAAGGAATGG-3ʹ
SNHG1 Reverse primer	5′-TAGAGGCAGACTGTCATCAGGAATACC-3ʹ
U6 Forward primer	5′-CTCGCTTCGGCAGCACA-3ʹ
U6 Reverse primer	5′-AACGCTTCACGAATTTGCGT-3ʹ
miR-641RT	5′-GTCGTATCCAGTGCAGGGTCCGAGGTATTCGCACTGGATACGACGAGGTG-3ʹ
miR-641Forward primer	5′-GCGCGAAAGACATAGGATAGAGT-3ʹ
miR-641Reverse primer	5′-AGTGCAGGGTCCGAGGTATT-3ʹ

### Cell transfection

SiRNAs targeting SNHG1, the miRNA control, miR-641, miR-641 inhibitory control, and miR-641 inhibitor were purchased by Gene Pharma (Shanghai, China). Si-NC (5′-UUCUCCGAACGUGUCACGUTT-3′), si-SNHG1-1 (5′-GAGCAAAUAAGGUGUAUAATT-3′), si-SNHG1-2 (5′-CCAGCACCUUCUAAATT-3′), and si-SNHG1-3 (5′-GGCCAUAGCUUUAAGAGAUTT-3′) served as control and si-SNHG1 sequences. The pcDNA-3.1-RRS1 plasmid was obtained from Shanghai GeneChem (China). Following the manufacturer's instructions, lipofectamine 2000 (Invitrogen) was used for cell transfection. sh-SNHG1 and negative control were synthesized by Obio (Shanghai, China).

### Western blotting

Cells, washed twice with phosphate-buffered saline (PBS), were fully lysed using RIPA, and protein quantification was performed using the BCA kit (Shanghai Epizyme Biomedical Technology, China). Protein samples were added to a quarter-volume 5× sodium dodecyl sulfate buffer solution, boiled for 10 min, separated by SDS-PAGE, and then transferred to a PVDF membrane (Because the molecular weights of the target proteins are very close, the length of the trimmed PVDF membrane is relatively short). After blocking the membranes with 5% skim milk and Tris-buffered saline in Tween-20 at 25 °C, they were incubated overnight at 4 °C with anti-GAPDH (1:1000), anti-*N*-cadherin (1:1000), anti-E-cadherin (1:1000), and anti-RRS1 (1:1000) antibodies (all purchased from Abcam, Cambridge, UK). Membranes were washed with TBS-T and then incubated with a horseradish peroxidase-labeled secondary antibody (1:1000; Abclonal, Woburn, MA, USA) for 1 h. Membranes were visualized with an enhanced chemiluminescence kit, and protein expression was measured using Image J v1.53t. GAPDH served as the internal control.

### Dual-luciferase reporter gene assay

The wild-type (WT) and corresponding mutated (MUT) sequences of *SNHG1* and *RRS1*, binding to *miR-641*, were synthesized and cloned into the pSI-check2 (Hanbio Biotechnology, Shanghai, China) reporter vector. MCF-7 and BT-549 cells were cultured in 24-well plates, and reporter vectors were co-transformed with *miR-641* mimics or NC using LipoFiter 3.0™ (Hanbio Biotechnology, Shanghai, China). After 48 h of cell culture, relative luciferase activity was measured using the dual-luciferase reporter assay system (Promega, Madison, WI, USA).

### Colony formation assay

Transfected cells were plated in 6-well plates at a density of 5 × 10^2^ cells per well and cultured in 10% FBS medium for 14 days. Colonies were fixed with 4% paraformaldehyde at 25 °C and stained with 600 mL of 5% crystal violet in each well for 20 min. Manual counting of colonies was performed after staining.

### Cell counting kit 8 (CCK-8) assay

To assess the impact of *SNHG1* on BC cells, 3 × 10^3^ cells were seeded in 96-well plates with five replicates per group. At 0, 1, 2, 3, and 4 days of culture, 10 μL of the CCK-8 kit (Solarbio, Beijing, China) was added to each well to detect cell proliferation and incubated at 37 °C for 120 min. Absorbance at 450 nm was measured using a microplate reader.

### Flow cytometry assay

Cells, digested with 0.25% trypsin and washed twice with PBS, were collected (1 × 10^6^ cells). Subsequently, 2.5 μL Annexin V-APC and 2.5 μL PI staining fluid were added to the cell suspension and incubated for 15 min. The rate of apoptosis was assessed using the CytoFLEX LX flow cytometer (Beckman Coulter, Brea, CA, USA).

### Transwell assays

To evaluate the impact of *SNHG1* on cell invasion and migration, transwell chambers coated with Matrigel (Corning, NY, USA) or uncoated were employed. Transfected cells (2 × 10^4^) were added to the upper chamber in 200 μL serum-free medium, while 600 μL 20% serum medium was placed in the bottom chamber. After 24 h cell culture, cells that migrated or invaded the membrane were fixed with methanol and stained with 0.5% crystal violet at 25 °C. Subsequently, cells were observed and counted under a microscope.

### RNA pulldown assays

BC cells were transfected with 100 nM biotin-labeled *miR-641* or negative control probes (GenePharma, Shanghai, China). After 48 h cultivation, 1 × 10^7^ cells were harvested and lysed. RNA pull-down experiments were conducted using the RNA Pull-Down Kit (BersinBio, Guangzhou, China). Streptavidin magnetic beads and cell lysates were mixed and incubated overnight at 4 °C with rotation, following the manufacturer's instructions. Subsequently, captured SNHG1 was purified with TRIzol and analyzed using qRT-PCR.

### In vivo experiments

Animal experiments were conducted with approval from the Qingdao University Laboratory Animal Welfare Ethics Committee (approval number: 202207BALB/cnude12202208082, date: 5 June 2022). All procedures adhered to relevant guidelines and regulations and were reported in accordance with ARRIVE guidelines.

BALB/c nude mice (4 weeks old) were supplied by Beijing Vital River Laboratory Animal Technology (China). Sh-SNHG1 or negative control MCF-7 cells (5 × 10^6^) were resuspended in a 200 μL mixture of 100 μL PBS with 100 μL Matrigel (Corning) and subcutaneously injected into the anterior part of mice (*n* = 5 per group). Tumor volume was calculated using the formula *V* = (length × width^2^)/2 every 3 days after tumor formation. On day 24, mice were euthanized using the cervical dislocation method under anesthesia (0.7% sodium pentobarbital), and tumors were harvested and weighed. Tumor tissues were fixed with paraformaldehyde, embedded in paraffin, and cut into 5-μm sections for immunohistochemistry (IHC). The primary antibody RRS1 (1:200, ab188161; Abcam) was used for IHC, with nuclei stained blue with hematoxylin and RRS1-positive level appearing brown or brownish yellow with diaminobenzidine (Supplementary Information).

### Statistical analysis

All experimental data were analyzed using SPSS v13. 0 software and GraphPad Prism v8.0 software. The data were presented as mean ± standard deviation (SD), and differences between groups were determined by Student's *t*-test. *P*-value < 0.05 was considered statistically significant.

## Results

### *SNHG1* expression is elevated in BC

**Figure 1:**
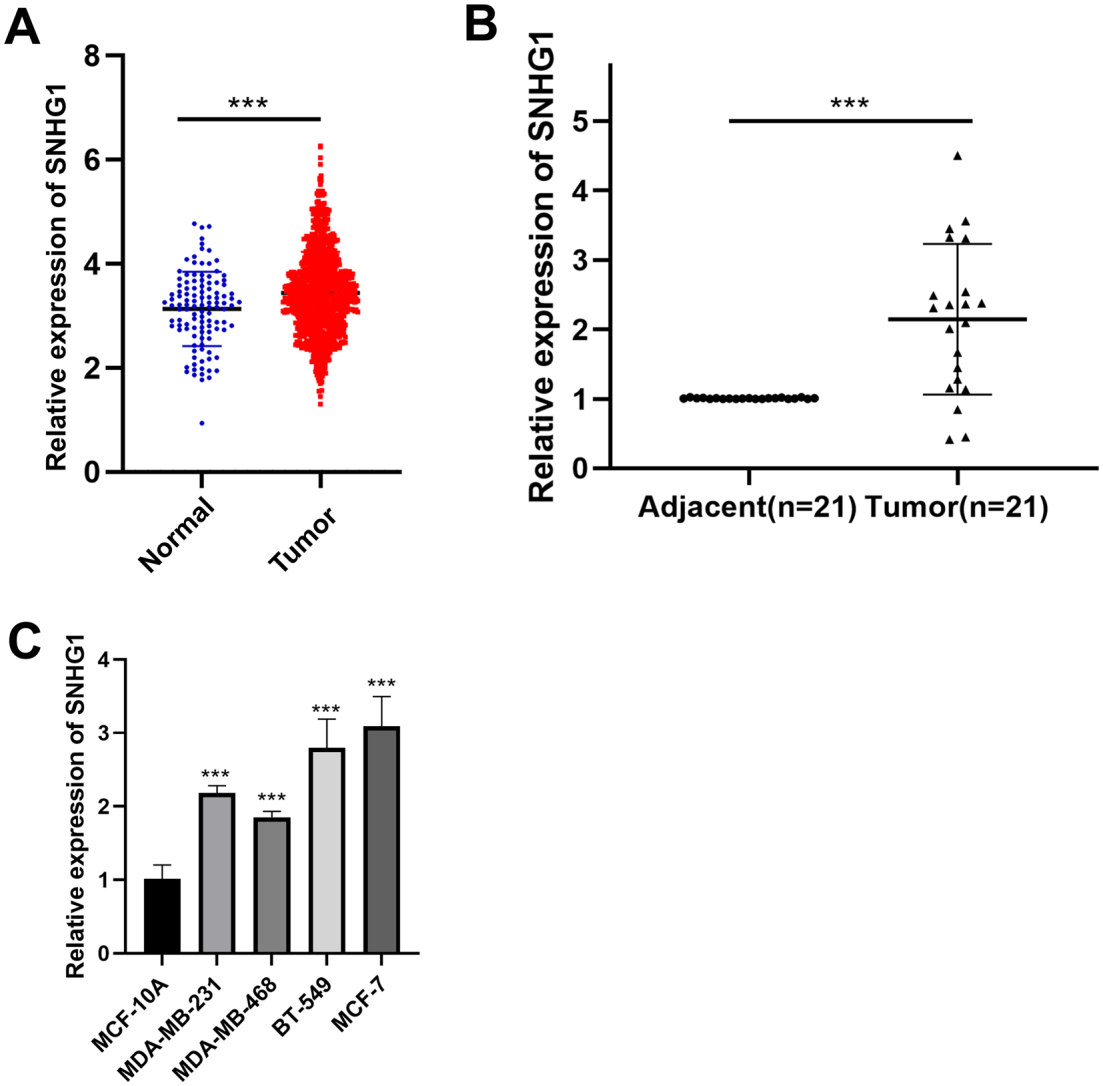
*SNHG1* is overexpressed in breast cancer (BC). (**A**) Comparison of *SNHG1* expression in human BC tissues based on the TCGA data set. (**B**) Expression of *SNHG1* in 21 paired BC tissues and normal tissues. (**C**) *SNHG1* expression in BC cell lines and the MCF-10A cell line. ***P < 0.001.

Upon analysis of the The Cancer Genome Atlas (TCGA) public database, it was observed that *SNHG1* expression was significantly elevated in BC compared to normal tissues (Fig. 1A). Using qRT-PCR, we further investigated *SNHG1* expression in BC tissues and found it to be notably higher than in paired normal tissues (Fig. 1B). Similarly, four human BC cell lines (MDA-MB-231, MDA-MB-468, MCF-7, and BT-549) expressed *SNHG1* at significantly higher levels than the MCF-10A cell line (Fig. 1C).

### Knockdown of *SNHG1* inhibited BC cell proliferation, migration, invasion, and induced apoptosis

**Figure 2:**
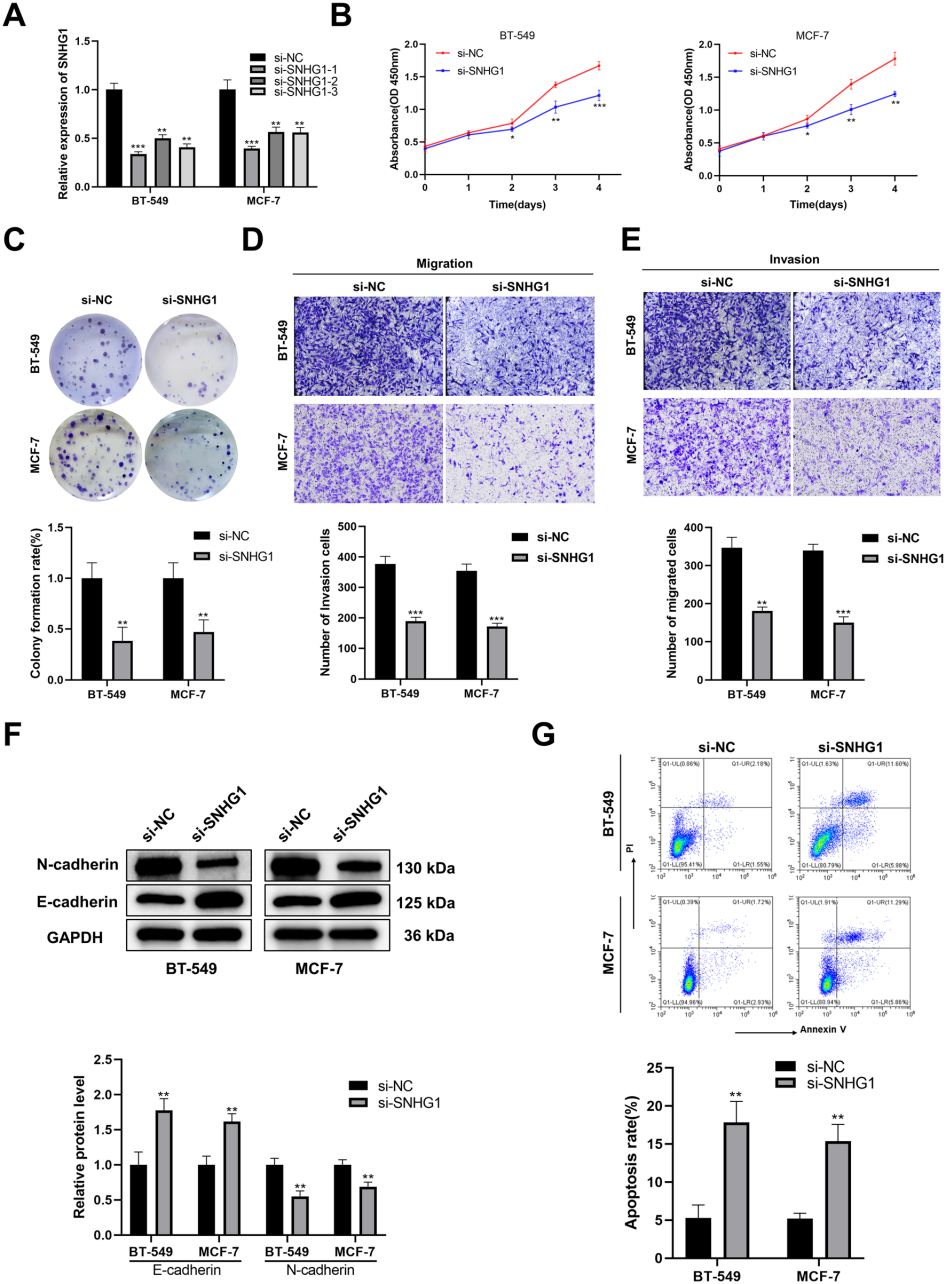
Knockdown of lncRNA *SNHG1* inhibited proliferation, migration, invasion, and induced apoptosis in BC. (**A**) Levels of *SNHG1* in BT-549 and MCF-7 after *SNHG1* interference. (**B**, **C**) CCK8 assay (**B**) and colony formation assay (**C**) are utilized to evaluate the effect of *SNHG1* on BC cell growth. (**D**, **E**) Transwell assays are used to assess cell migration (**D**) and invasion (**E**) ability. (**F**) The levels of EMT-related proteins are analyzed by Western blotting. (**G**) Flow cytometry assay applied to identify cell apoptosis. **P < 0.01; ***P < 0.001.

To investigate the impact of *SNHG1* on BC cells in vitro, we selected BT-549 and MCF-7, which exhibited relatively high *SNHG1* expression, and used siRNA to interfere with *SNHG1* expression. qRT-PCR analysis revealed that *SNHG1* expression decreased in cells transfected with three types of *SNHG1* siRNA compared to the control group, with the most effective knockdown observed with si-SNHG1-1 (Fig. 2A). Consequently, si-SNHG1-1 was employed in subsequent research. The CCK8 assay and colony formation assay indicated that *SNHG1* knockdown significantly inhibited BC cell proliferation and reduced clone formation (Fig. 2B,C). Additionally, transwell assays were used to assess the effects of *SNHG1* on BC cell migration and invasion, revealing a marked decrease in both abilities following *SNHG1* knockdown (Fig. 2D,E). EMT, closely associated with tumor invasion and migration, was analyzed by Western blot. The level of EMT-related protein N-cadherin significantly decreased after *SNHG1* silencing, while the level of E-cadherin increased (Fig. 2F). Flow cytometry analysis demonstrated that *SNHG1* knockdown induced BC cell apoptosis (Fig. 2G). These findings indicate that the lncRNA *SNHG1* plays a crucial role in regulating BC proliferation, migration, invasion, and apoptosis.

### *SNHG1* regulates the expression of *miR-641*

**Figure 3:**
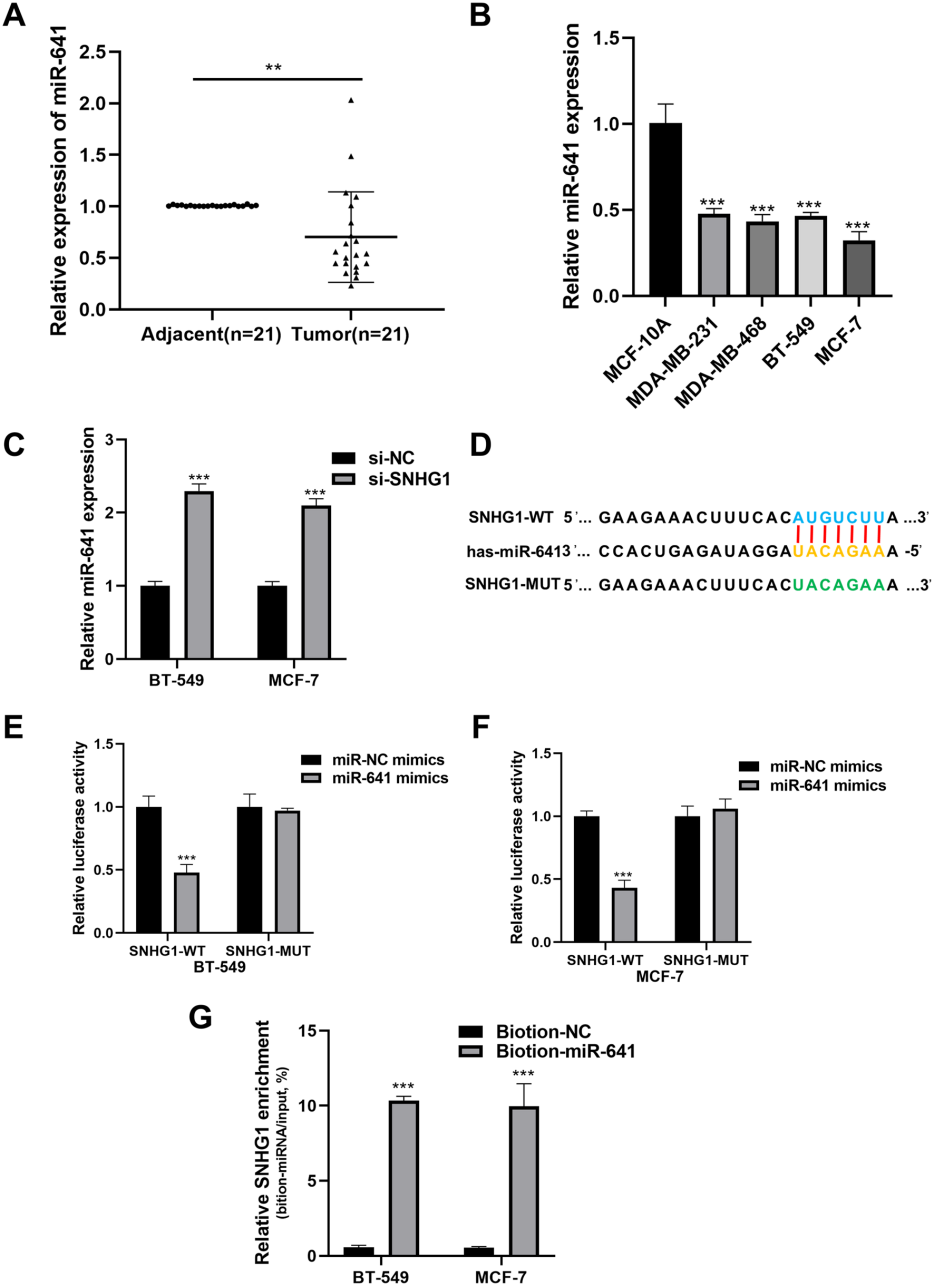
*SNHG1* targets *miR-641*. (**A**) The level of *miR-641* in 21 pairs of BC tissues and normal tissues. (**B**) The level of *miR-641* in BC cells detected by qRT-PCR. (**C**) Relative levels of *miR-641* in MCF-7 and BT-549 cells transfected with si-SNHG1 or si-NC. (**D**) Binding site between *SNHG1* and *miR-641*. (**E**, **F**) In BC cells BT-549 (**E**) and MCF-7 (**F**), the dual-luciferase reporter gene assay is used to detect the interaction between *SNHG1* and *miR-641*. (**G**) Enrichment of *SNHG1* pulled down by biotin-*miR-641* or negative control. **P < 0.01; ***P < 0.001.

An increasing number of studies have indicated that lncRNAs can bind to miRNAs, acting as competing endogenous RNAs (ceRNAs)^21^. The level of *miR-641* in BC tissues and cells was assessed using qRT-PCR, revealing downregulation in BC tissues and cells (Fig. 3A,B). In BT-549 and MCF-7 cells, silencing *SNHG1* expression led to an increase in the level of *miR-641* (Fig. 3C). The sequence complementarity between *SNHG1* and *miR-641* was predicted using Starbase v2.0 (http://starbase.sysu.edu.cn/) (Fig. 3D). Subsequently, the dual-luciferase reporter gene assay was conducted to validate the binding relationship between *SNHG1* and *miR-641*. *MiR-641* mimics significantly reduced the luciferase activity of SNHG1-WT, but did not affect the luciferase activity of SNHG1-MUT (Fig. 3E,F). Additionally, RNA pull-down experiments demonstrated a significant increase in the ratio of endogenous *SNHG1* pulled down by biotin-labeled *miR-641* (Fig. 3G). In conclusion, our findings demonstrate that *SNHG1* can directly bind to *miR-641*, suggesting that the oncogenic mechanism of *SNHG1* may involve the negative regulation of *miR-641*.

### RRS1 is directly targeted by *miR-641* and upregulated in BC

**Figure 4:**
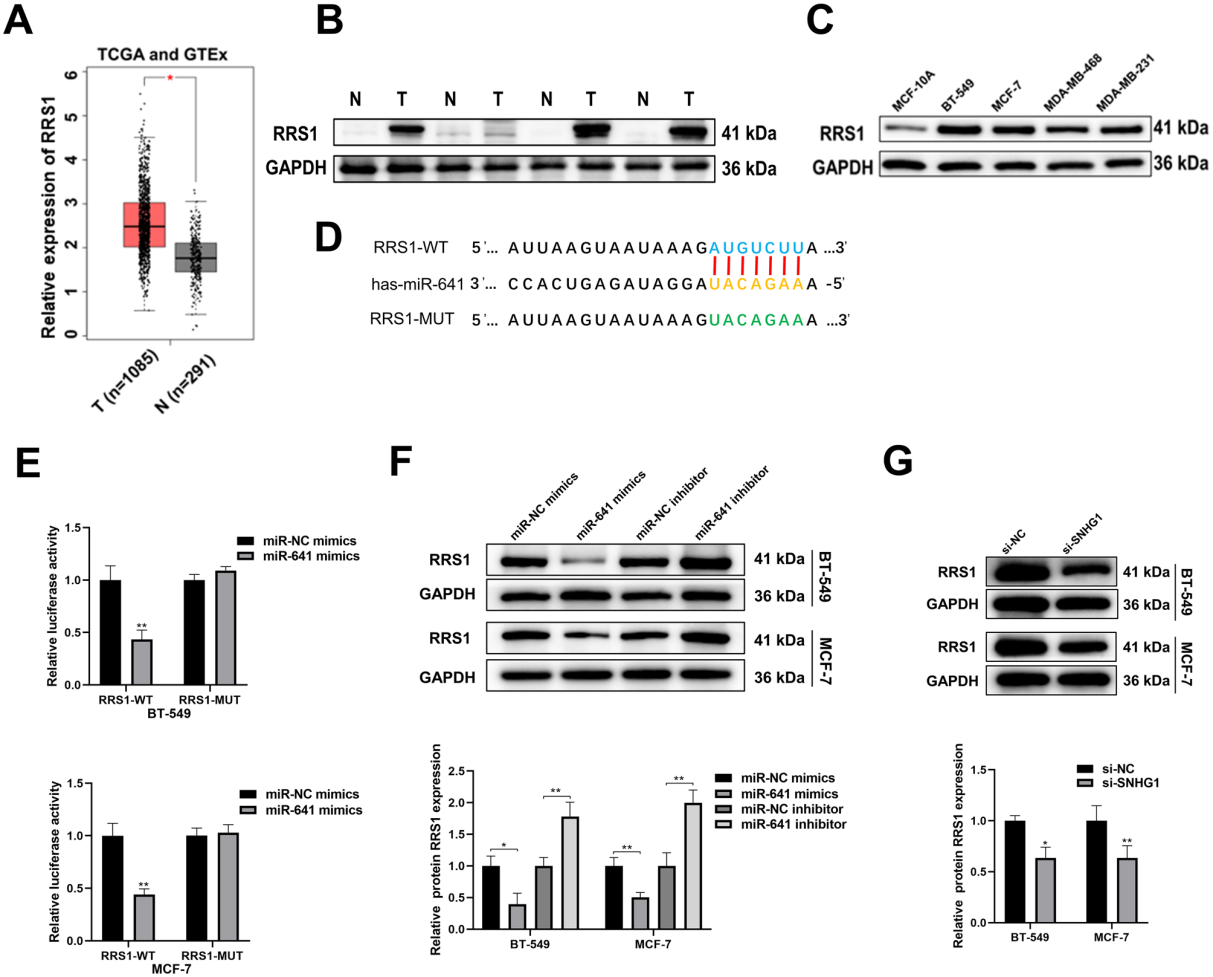
RRS1 is overexpressed in BC and targeted by *miR-641*. (**A**) GEPIA confirms the obvious overexpression of *RRS1* in BC tissues based on the TCGA cohort. (**B**) The level of RRS1 in four pairs of BC tissues was analyzed by Western blot. (**C**) Protein levels of RRS1 in BC cells. (**D**) Binding sites of *RRS1* and *miR-641*. (**E**) *MiR-641* specifically targets RRS1. (**F**) Relative level of RRS1 protein after *miR-641* overexpression or knockdown. (**G**) Relative level of RRS1 after knockdown of *SNHG1*. *P < 0.05; **P < 0.01.

To further investigate the mechanism of *SNHG1* action in BC, we utilized the targetScan database (https://www.targetscan.org/vert_72/) to identify *miR-641*'s downstream target gene *RRS1*. Analysis using the GEPIA database (http://gepia.cancer-pku.cn/), revealed elevated *RRS1* mRNA levels in BC tumor tissues compared to normal tissues in the TCGA cohort (Fig. 4A). Similarly, Western blot results demonstrated a significant increase in RRS1 protein level in cancer tissues compared to BC-paired normal tissues (Fig. 4B). Moreover, RRS1 was found to be upregulated in BC cell lines (Fig. 4C). Subsequent dual-luciferase reporter gene assays confirmed the binding of *miR-641* to the 3'-UTR of *RRS1* as predicted by TargetScan (Fig. 4D,E). *MiR-641* upregulation markedly inhibited RRS1 protein level, while *miR-641* downregulation increased RRS1 protein level (Fig. 4F). We further explored the regulation of RRS1 level by *SNHG1*. After silencing *SNHG1*, the protein level of RRS1 significantly decreased (Fig. 4G). These results indicate that *RRS1* is a direct target of *miR-641* and is regulated by *SNHG1*.

### Down-regulation of *miR-641* or upregulation of RRS1 reverses the effect of *SNHG1* silencing on BC cell

**Figure 5:**
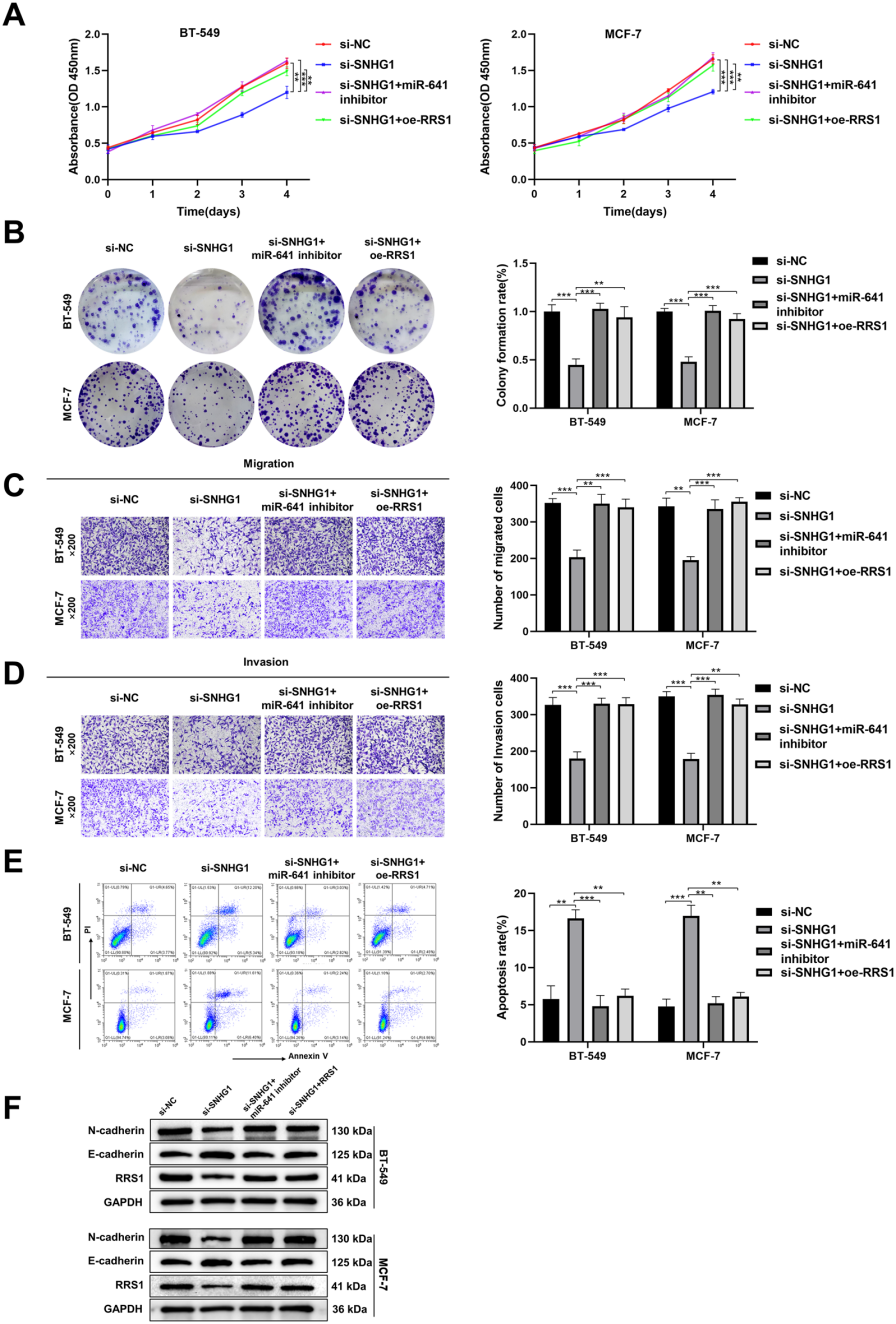
Altering the expression of *miR-641* and RRS1 reversed the effect of silencing *SNHG1*. (**A**, **B**) Cell growth was measured using the CCK-8 assay (**A**) and the colony formation assay (**B**). (**C**, **D**) Transwell assay was used to detect cell migration (**C**) and invasion (**D**). (**E**) Detection of cell apoptosis by flow cytometry. (**F**) The levels of RRS1 and EMT-related proteins detected by western blotting. **P < 0.05; ***P < 0.001.

To explore whether *SNHG1* mediates the impact of *miR-641* and RRS1 on BC growth, migration, invasion, and apoptosis, rescue experiments were conducted by down-regulating *miR-641* or upregulated *RRS1* in the context of *SNHG1* silencing. Silencing *SNHG1* inhibited the growth, migration, and invasion, as well as promoted apoptosis of BC cells, but these effects were partially reversed by down-regulation of *miR-641* or restoration of RRS1 level (Fig. 5A–E). Additionally, transfection with the *miR-641* inhibitor or upregulation of RRS1 also counteracted the changes in EMT-related proteins and RRS1 level observed after silencing *SNHG1* (Fig. 5F). Overall, these findings further underscore that *SNHG1* promotes the progression of BC cells through the *miR-641*/*RRS1* axis.

### *SNHG1* intervention inhibits the growth of BC tumors in vivo

**Figure 6:**
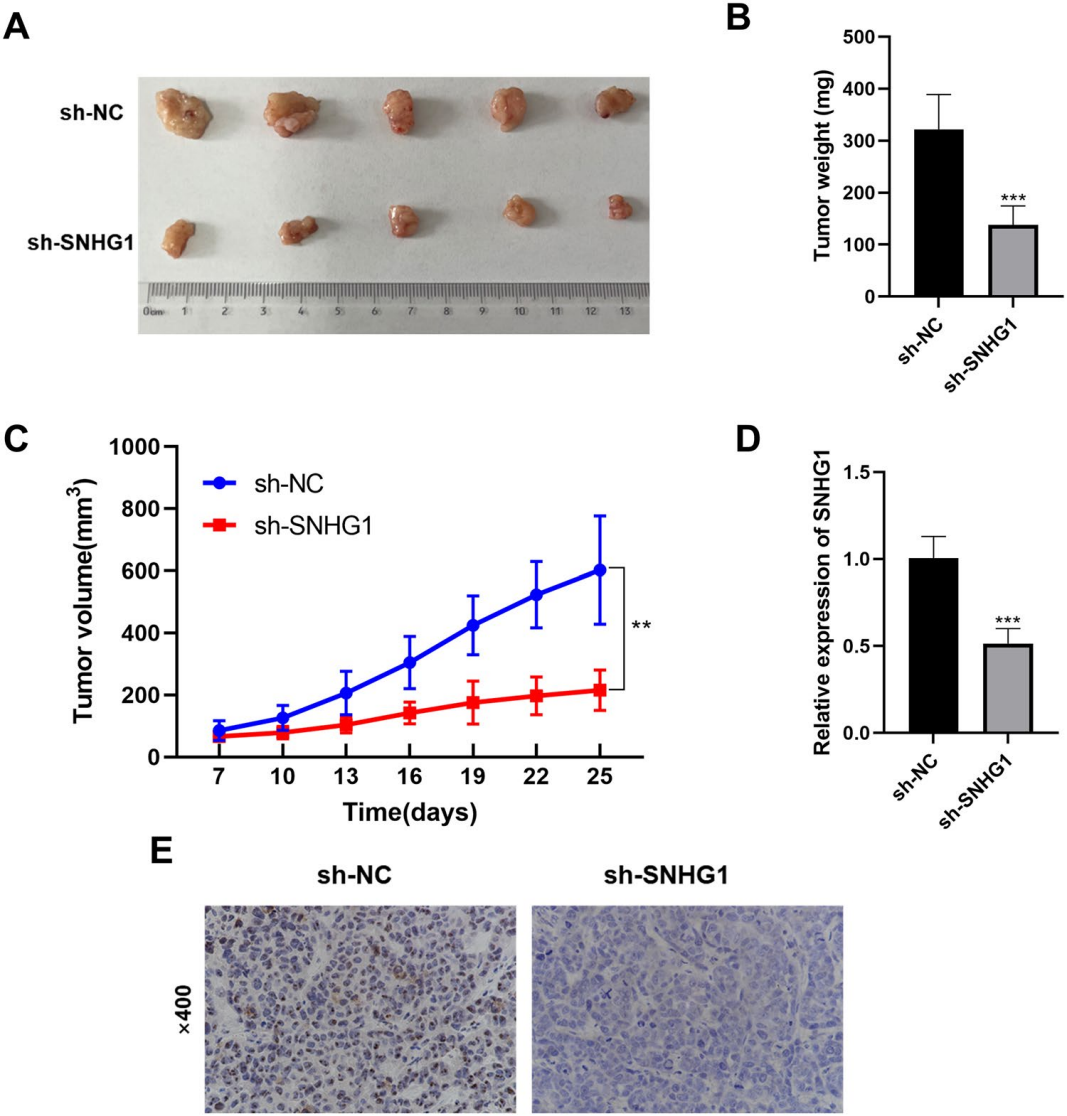
Down-regulation of *SNHG1* inhibits the progression of BC in vivo. (**A**) Tumor size. (**B**) Tumor weight. (**C**) Tumor growth curve. (**D**) The level of *SNHG1* in xenograft tumors. (**E**) The level of RRS1 in xenograft tumors detected by IHC. **P < 0.01; ***P < 0.001.

To delve into the role of *SNHG1* in tumorigenesis in vivo, *SNHG1*-stable-knockdown MCF-7 cells were subcutaneously injected into nude mice to establish a xenograft mouse model. It was observed that tumor size, weight, and growth curves were significantly suppressed after *SNHG1* silencing (Fig. 6A–C). The xenograft tumors derived from the sh-NC group exhibited higher *SNHG1* levels (Fig. 6D). Additionally, IHC results demonstrated a decrease in RRS1-positive cells in tumor tissues following *SNHG1* knockdown (Fig. 6E). The downregulation of *SNHG1* can effectively inhibit the growth of BC tumors in vivo.

## Discussion

BC is a highly heterogeneous disease, and the overall treatment effect is not ideal. Therefore, exploring new treatment methods to reduce BC mortality remains the focus of public attention^22,23^. The dysregulation of lncRNA is strongly associated with the development of various malignant tumors, including BC^24^. Consequently, an increasing number of studies are concentrating on investigating the functions and regulation of lncRNAs to identify new targets for cancer diagnosis and treatment.

*SNHG1* comprises 8 small nucleolar RNAs with 11 exons^14^. Currently, there are limited studies on the function and mechanism of *SNHG1* in BC. Previous research has indicated that *SNHG1* is highly expressed in hepatocellular carcinoma, where it mediates sorafenib resistance through activating the AKT pathway and is linked to poor prognosis^25^. Similarly, our experiments revealed up-regulation of *SNHG1* in BC tissues and cells. Moreover, accumulating evidence suggests that lncRNAs play a crucial role in regulating various biological processes in BC, including proliferation and metastasis. For instance, Xue et al. demonstrated that lncRNA *AC073352.1* binds to YBX1 to promote BC metastasis and angiogenesis^8^. In prostate cancer, SNHG1 promotes EMT processes by competitively binding to hnRNPL, preventing E-cadherin translation^16^. Our in vivo results indicated that the downregulation of *SNHG1* suppressed BC progression. Consistent with our findings, interference with lncRNA *SBF2-AS1* inhibited tumor growth in the BC xenograft tumor model^26^. In vitro, silencing *SNHG1* expression significantly reduced BC cell proliferation, EMT, invasion, and migration, while promoting apoptosis.

Multiple studies have unveiled that lncRNA, operating as a ceRNA by sequestering miRNAs, plays a crucial role in regulating the biological functions of tumors^27,28^. Previous investigations have indicated the presence of *SNHG1* in both the cytoplasm and nucleus of BC cells^29^. According to recent research, *MCM3AP-AS1* acts as a ceRNA in hepatocellular carcinoma, functioning as a sponge for *miR-194* to enhance *FOXA1* expression^30^. Another study has identified *SNHG1* as a ceRNA that promotes gastric cancer cell proliferation by modulating the *miR-140*/ADAM10 axis^31^. Mechanistically, we hypothesized that *SNHG1* operates as a ceRNA through miRNA sequestration. Initially, bioinformatics analysis revealed a potential binding sequence between *SNHG1* and *miR-641*. Subsequent dual-luciferase reporter gene assays and RNA pull-down assays confirmed that *SNHG1* targets *miR-641*. Liang et al. previously discovered that *miR-641*, down-regulated in glioma, acts as a sponge for COX10-AS1^19^. Moreover, studies have indicated the regulatory role of *miR-641* in the BC process^32^. Rescue experiments demonstrated that a *miR-641* inhibitor partially reverses the inhibitory effects of *SNHG* knockdown on BC proliferation, migration, invasion, and EMT processes.

We comprehensively investigated the mechanism by which *miR-641* regulates RRS1. Our data demonstrated that *miR-641* can modulate the level of downstream RRS1, as confirmed by the dual-luciferase reporter gene assay. *RRS1* functions as an oncogene in cancer, with *RRS1* gene mutation and abnormal expression promoting tumor growth and metastasis^33^. Silencing RRS1 inhibits EMT, migration, and invasion in BC cells, and RRS1 affects BC progression via the RPL11-c-Myc-SNAIL axis^20^. Down-regulation of *SBF2-AS1* and up-regulation of *miR-143* can inhibit proliferation and promote apoptosis in BC cells by suppressing RRS1 level^26^. The TCGA database and analysis of four pairs of BC tumor tissues revealed an elevated *RRS1* level. In this study, we discovered that *SNHG1* regulates BC cell proliferation, migration, invasion, EMT, and apoptosis processes. *SNHG1* modulates *RRS1* in BC cells by competitively binding to *miR-641*, and the overexpression of *RRS1* can restore the cellular functions mediated by *SNHG1*. Moreover, ribosome biosynthesis is one of the major functions of RRS1, and whether SNHG1 can mediate BC ribosome biosynthesis and its downstream molecular mechanisms through RRS1 needs to be thoroughly investigated.

## Conclusion

**Figure 7:**
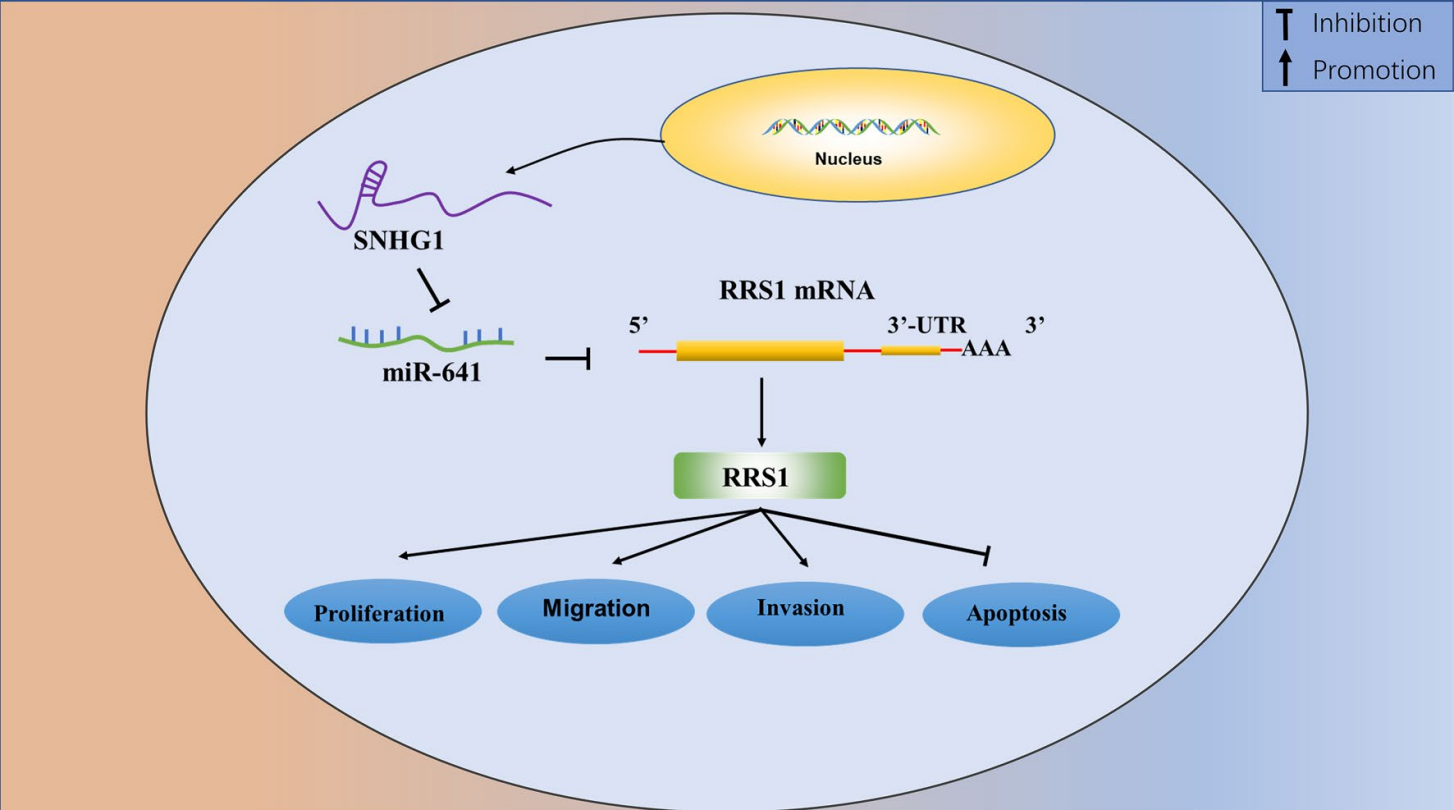
SNHG1 promoted BC progression via modulating the miR-641/RRS1 axis.

This study marks the first investigation into the molecular mechanism involving *SNHG1*, *miR-641*, and *RRS1*. Our findings confirmed the binding interaction between *SNHG1* and *miR-641*, establishing that the silencing of *SNHG1* could inhibit EMT, proliferation, migration, and invasion while promoting apoptosis in BC cells. This effect was achieved through the inhibition of the *miR-641*/*RRS1* axis **(**Fig. 7**)**. Consequently, our study provides new insights for molecularly targeted therapy in BC.

### Supplementary Information

Supplementary Information.

## Data Availability

Datasets on expression levels of SNHG1 and RRS1 from BC patients were obtained from The Cancer Genome Atlas (TCGA; The Cancer Genome Atlas Program—NCI).

## Abbreviations

LncRNA: Long non-coding RNA
BC: Breast cancer
SNHG1: Small nucleolar RNA host gene 1
EMT: Epithelial-mesenchymal transition
MicroRNAs: MiRNAs
3ʹ-UTR: 3ʹ-Untranslated region
RRS1: Regulator of ribosome synthesis 1
qRT-PCR: Quantitative reverse-transcription polymerase chain reaction
PBS: Phosphate buffered saline
CCK-8: Cell counting kit
TCGA: The Cancer Genome Atlas
ceRNA: Competing endogenous RNA
IHC: Immunohistochemistry
