# Supplementary material for: Long noncoding RNA SNHG1 promotes breast cancer progression by regulating the miR-641/RRS1 axis
Source: Sci Rep. 2024 Feb 8;14:3265. doi: 10.1038/s41598-024-52953-0 (PMC10853250; doi:10.1038/s41598-024-52953-0)
Supplement: Supplementary file 1 — Supplementary Information. [file 41598_2024_52953_MOESM1_ESM.pdf]

Note: All wb images in the article are from Replicate1

si-NC si-SNHG1

N-cadherin 130kDa

E-cadherin 125kDa

GAPDH 36kDa

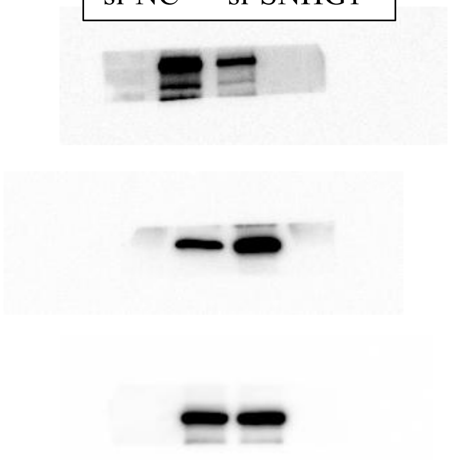

Fig.2F BT-549 cells Replicate1

si-NC si-SNHG1

N-cadherin 130kDa

E-cadherin 125kDa

GAPDH 36kDa

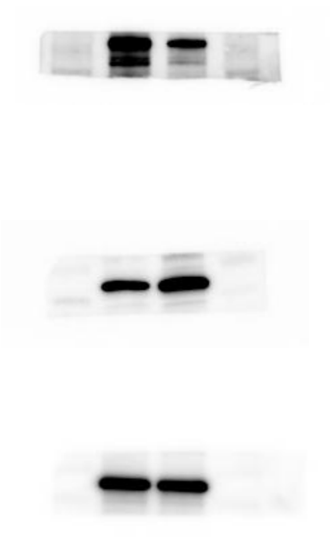

Fig.2F MCF-7 cells Replicate1

si-NC si-SNHG1

N-cadherin 130kDa

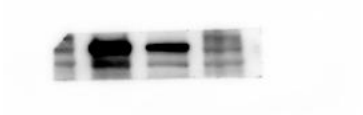

E-cadherin 125kDa

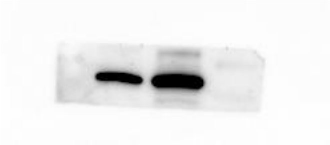

GAPDH 36kDa

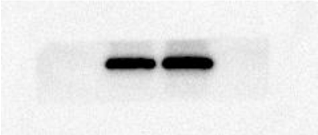

Fig.2F BT-549 cells Replicate2

---

si-NC si-SNHG1

N-cadherin 130kDa

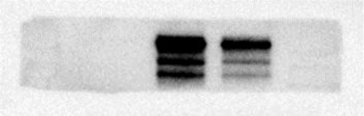

E-cadherin 125kDa

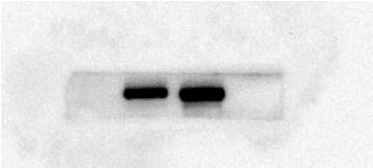

GAPDH 36kDa

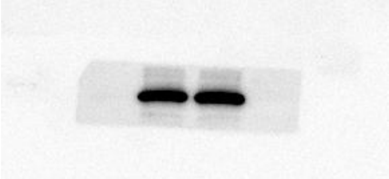

Fig.2F MCF-7 cells Replicate2

si-NC si-SNHG1

N-cadherin 130kDa

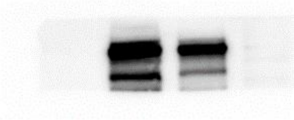

E-cadherin 125kDa

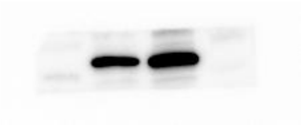

GAPDH 36kDa

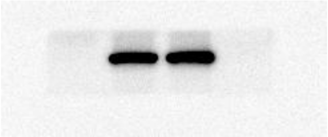

Fig.2F BT-549 cells Replicate3

---

si-NC si-SNHG1

N-cadherin 130kDa

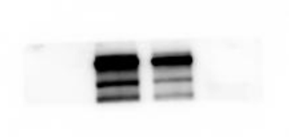

E-cadherin 125kDa

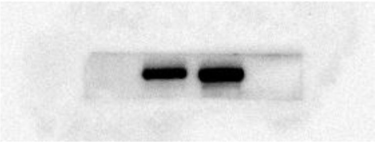

GAPDH 36kDa

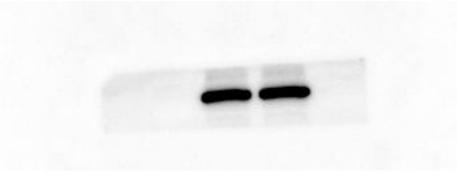

Fig.2F MCF-7 cells Replicate3

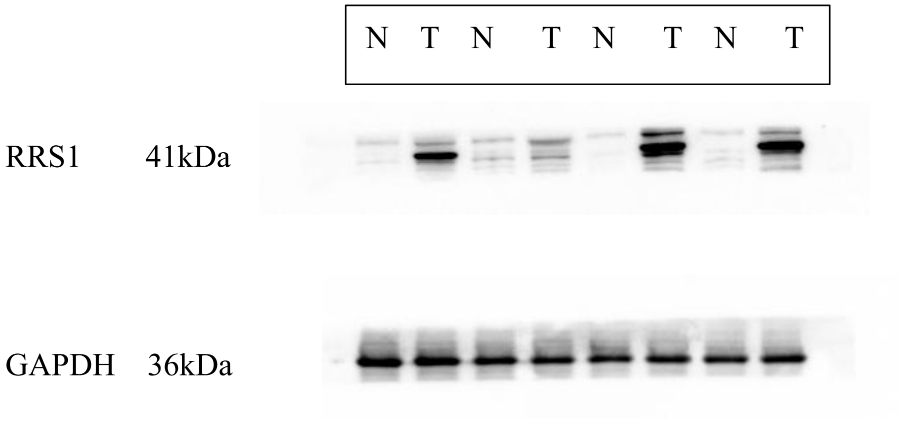

Fig.4B Replicate1

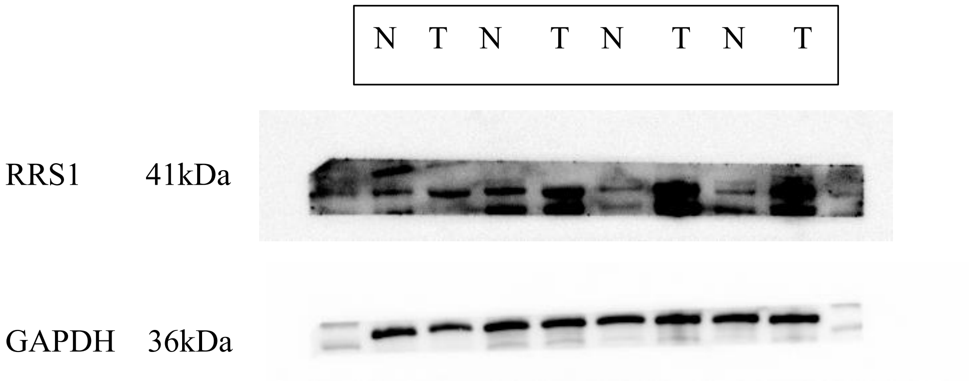

Fig.4B Replicate2

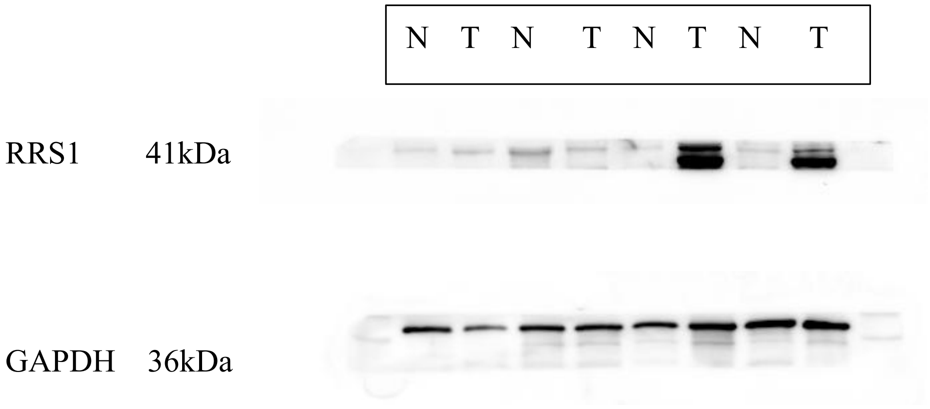

Fig.4B Replicate3

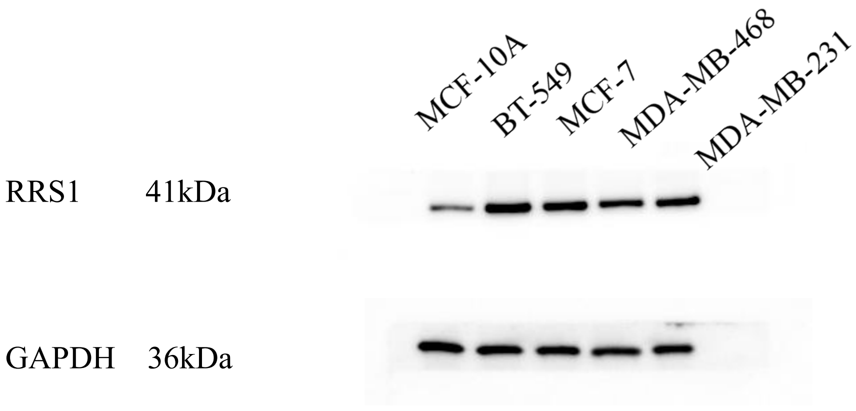

Fig.4C Replicate1

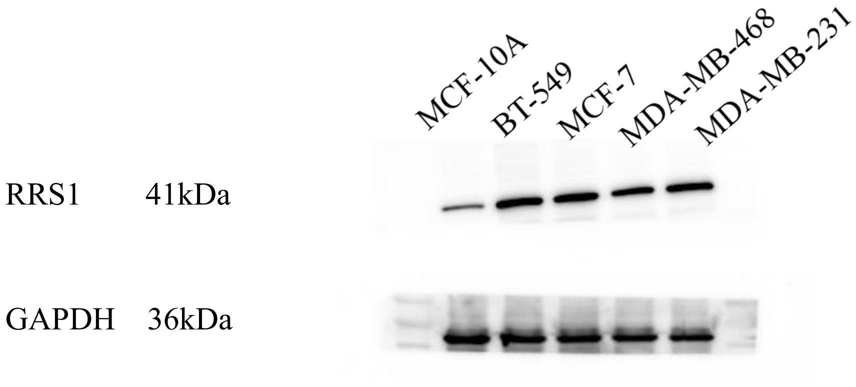

Fig.4C Replicate2

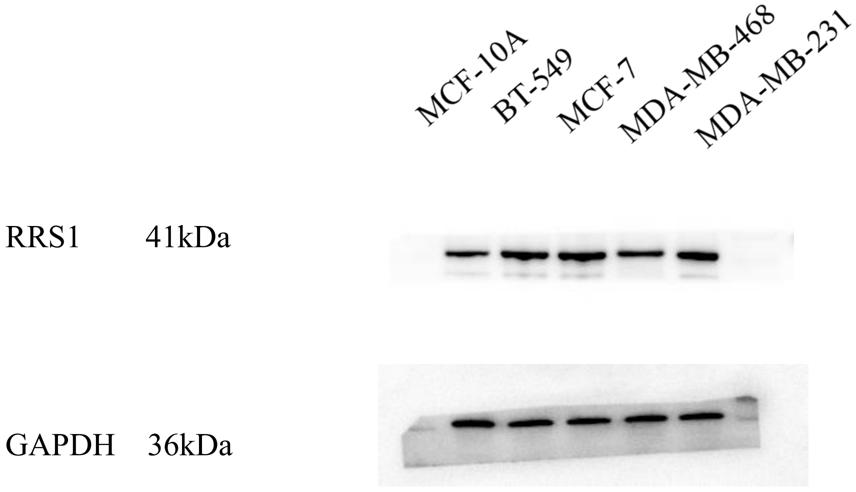

Fig.4C Replicate3

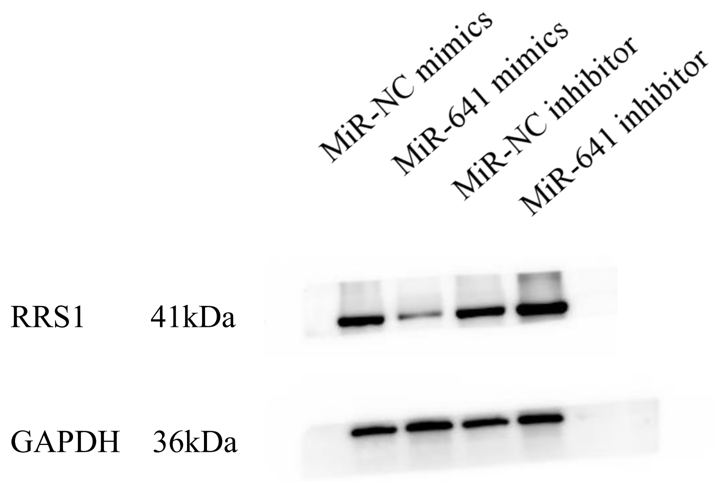

Fig.4F BT-549 cells Replicate1

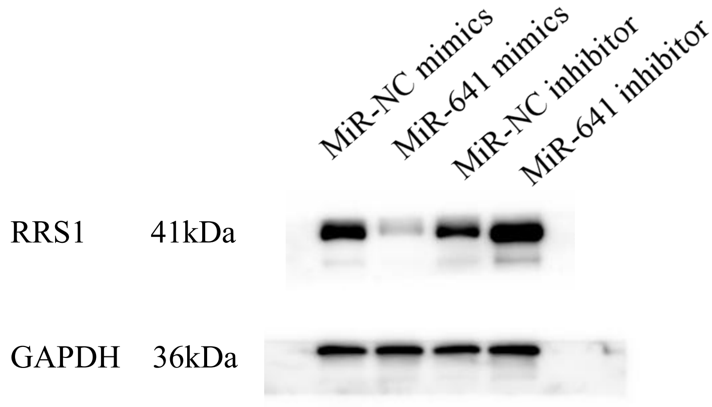

Fig.4F BT-549 cells Replicate2

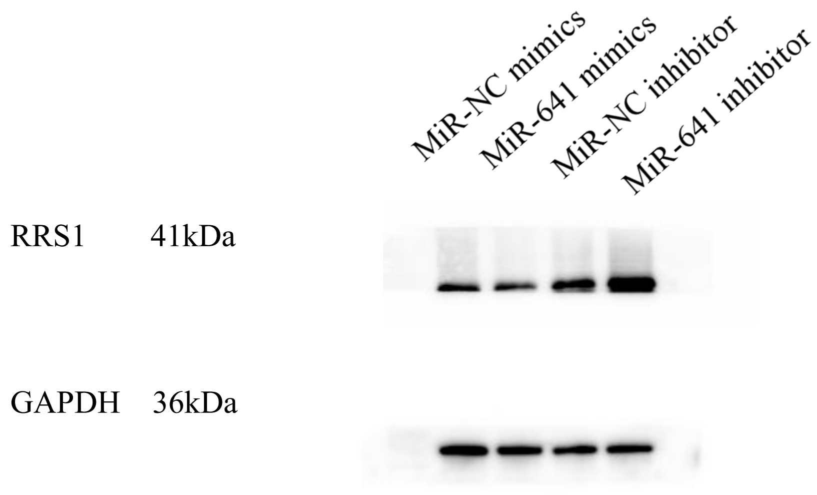

Fig.4F BT-549 cells Replicate3

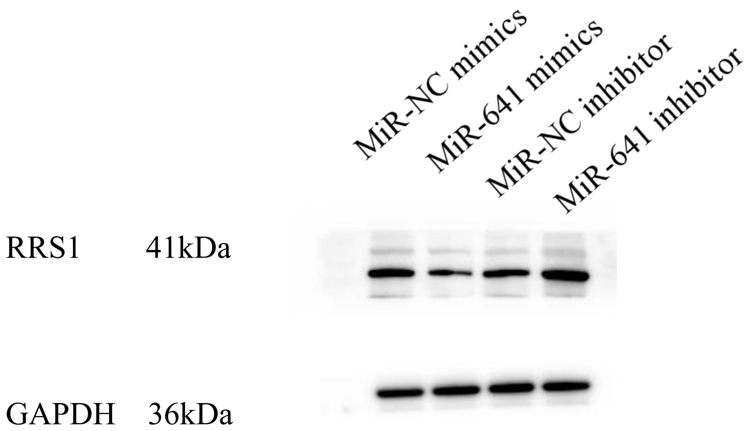

Fig.4F MCF-7 cells Replicate1

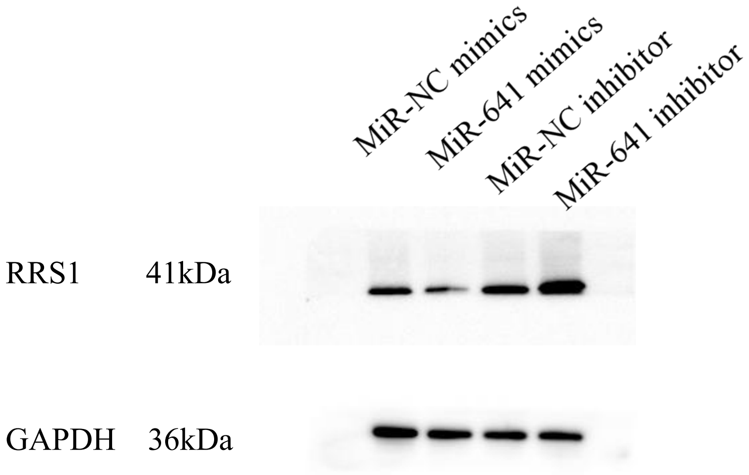

Fig.4F MCF-7 cells Replicate2

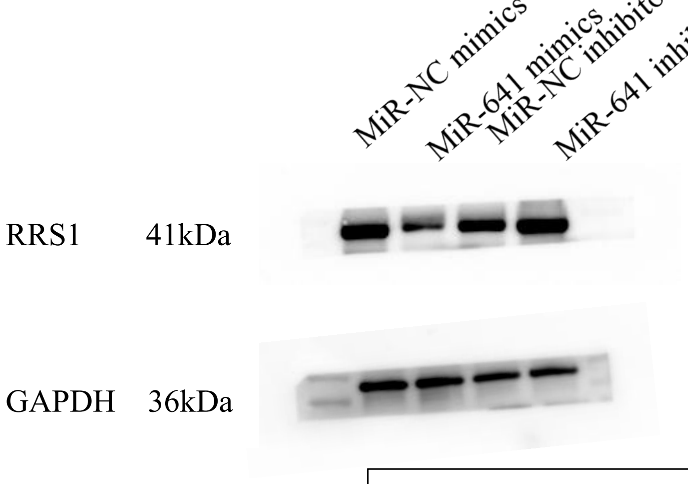

Fig.4F MCF-7 cells Replicate3

si-NC si-SNHG1

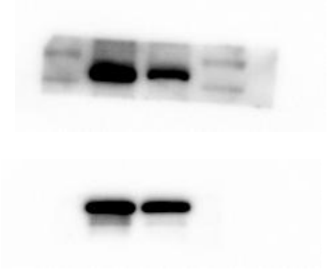

RRS1 41kDa

GAPDH 36kDa

Fig.4G BT-549 cells Replicate1

---

si-NC si-SNHG1

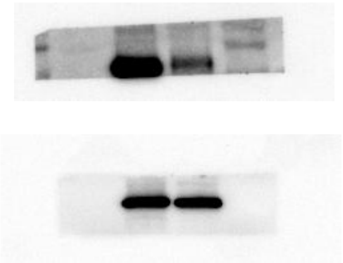

RRS1 41kDa

GAPDH 36kDa

Fig.4G BT-549 cells Replicate2

---

si-NC si-SNHG1

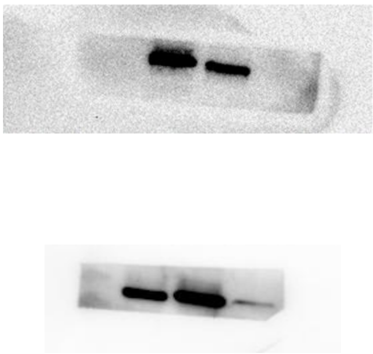

RRS1 41kDa

GAPDH 36kDa

Fig.4G BT-549 cells Replicate3

si-NC si-SNHG1

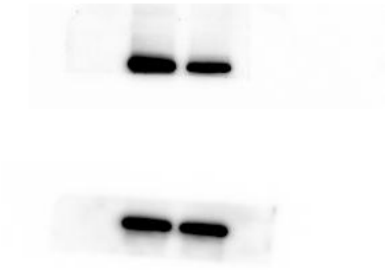

RRS1 41kDa

GAPDH 36kDa

Fig.4G MCF-7 cells Replicate1

---

si-NC si-SNHG1

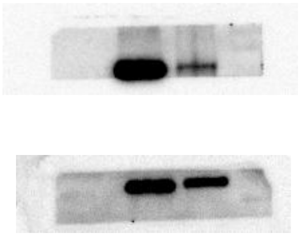

RRS1 41kDa

GAPDH 36kDa

Fig.4G MCF-7 cells Replicate2

---

si-NC si-SNHG1

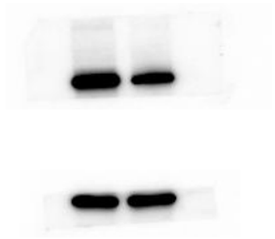

RRS1 41kDa

GAPDH 36kDa

Fig.4G MCF-7 cells Replicate3

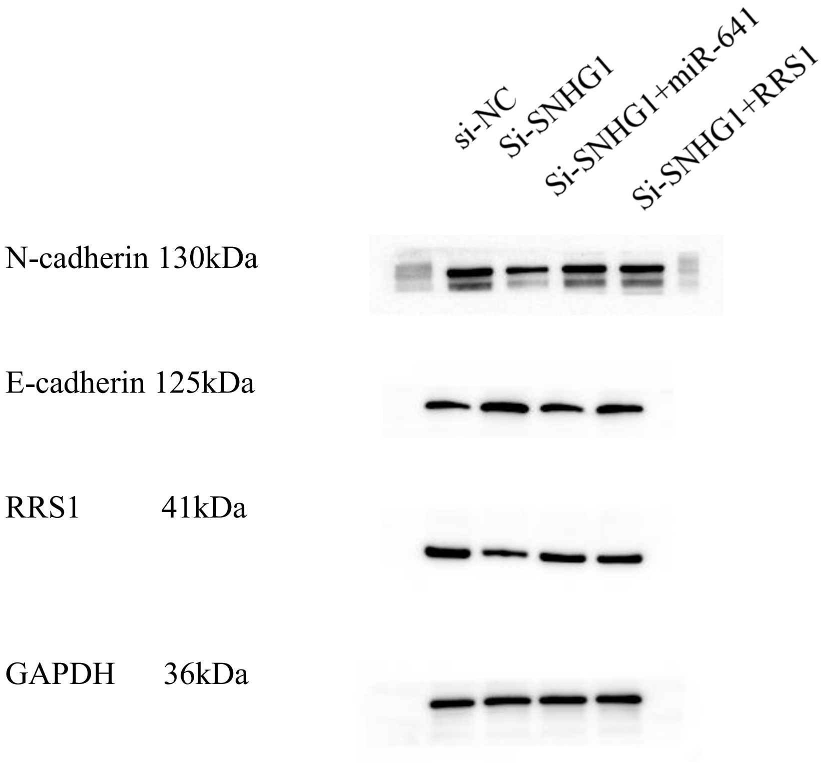

Fig.5F BT-549 cells Replicate1

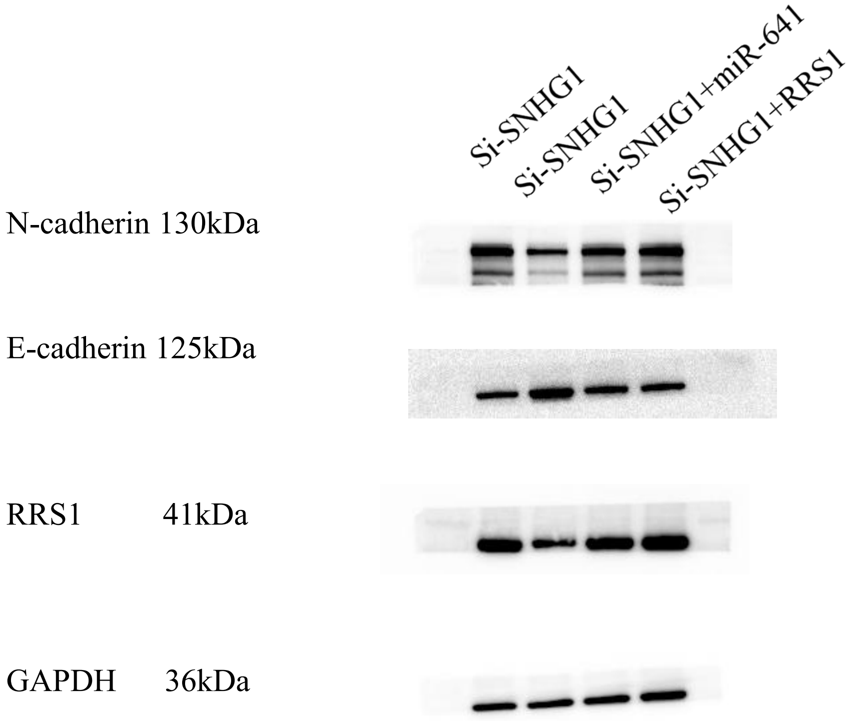

Fig.5F BT-549 cells Replicate2

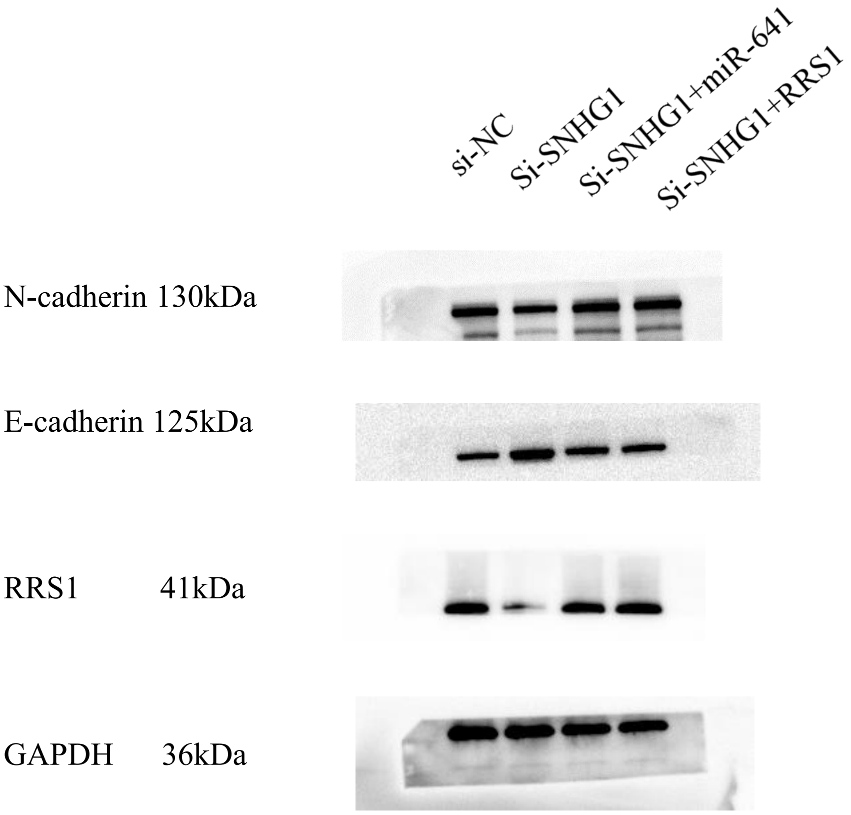

Fig.5F BT-549 cells Replicate3

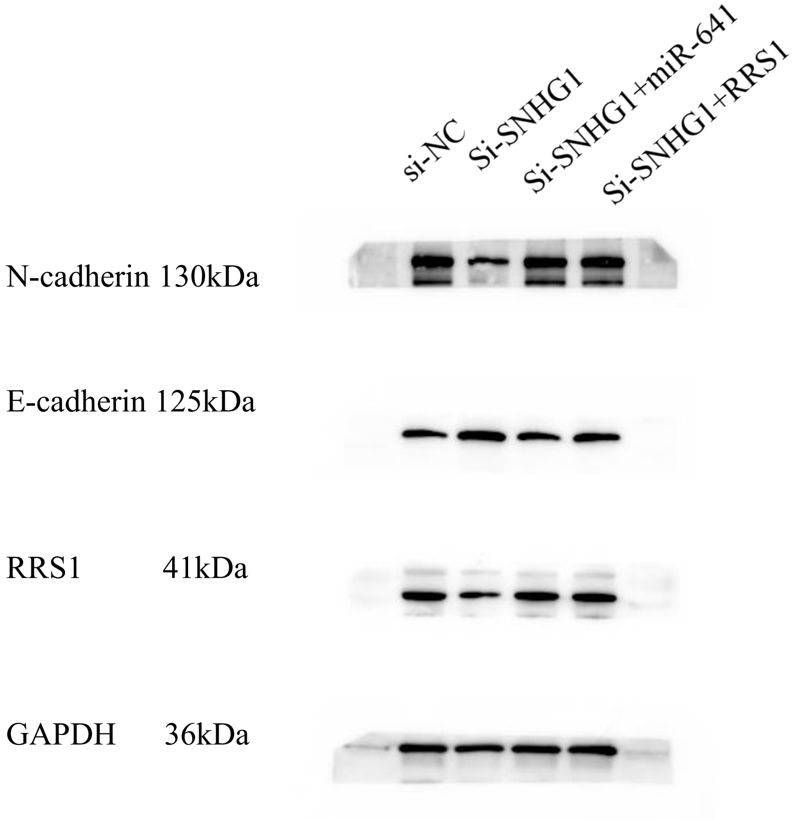

Fig.5F MCF-7 cells Replicate1

si-NC Si-SNHG1 Si-SNHG1+miR-641 Si-SNHG1+RRS1

N-cadherin 130kDa

E-cadherin 125kDa

RRS1 41kDa

GAPDH 36kDa

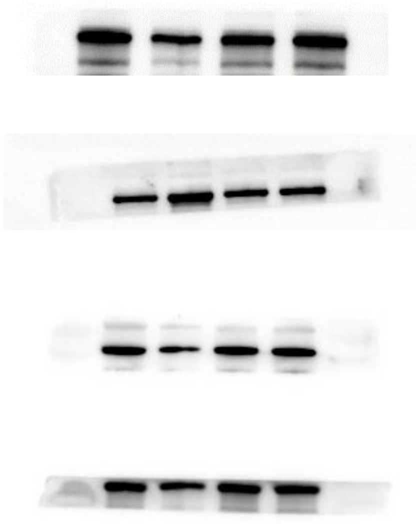

Fig.5F MCF-7 cells Replicate2

---

si-NC Si-SNHG1 Si-SNHG1+miR-641 Si-SNHG1+RRS1

N-cadherin 130kDa

E-cadherin 125kDa

RRS1 41kDa

GAPDH 36kDa

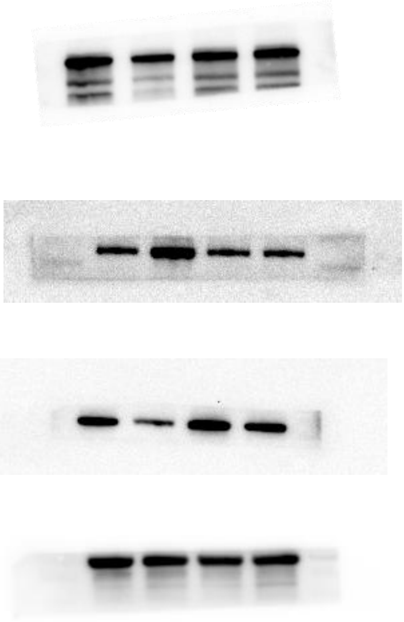

Fig.5F MCF-7 cells Replicate3
